# Blastocystis species in hepatic abscesses: innocent bystander or hidden culprit? A case report and literature review

**DOI:** 10.1099/acmi.0.001163.v4

**Published:** 2026-07-22

**Authors:** Si Qi Cao, Hasan Hamze, Richard T. Lester

**Affiliations:** 1 Department of Undergraduate Medicine, Faculty of Medicine, The University of British Columbia, 6200 University Blvd, Vancouver, BC V6T 1Z4, Canada; 2 Department of Pathology and Laboratory Medicine, The University of British Columbia, 2211 Wesbrook Mall, Vancouver, BC V6T 1Z7, Canada; 3 Department of Medicine, Division of Infectious Diseases, Faculty of Medicine, 328C, Heather Pavilion, VGH 2733 Heather Street, Vancouver, BC V5Z 3J5, Canada

**Keywords:** *Blastocystis *spp., liver abscess, pathogenicity, parasitology

## Abstract

*Blastocystis hominis* is a ubiquitous intestinal parasite commonly detected in human stools. Queries about the pathogenic nature of *B. hominis* in immunocompetent individuals have been raised with conflicting reports about the clinical significance of detecting the parasites in various specimens. Reports have documented invasive intestinal infections and the detection of *Blastocystis* species in both a splenic cyst and a hepatic abscess. We present the case of a 65-year-old man with a history of recent travel to a tropical country who was found to have multiple hepatic abscesses. Analysis of the hepatic abscess fluid confirmed the presence of *Entamoeba histolytica* and *Blastocystis* species, and, to our knowledge, this is the second reported case of the same findings. Overall, the clinical and microbiological picture was most consistent with amoebic liver abscess secondary to *E. histolytica*; however, the presence of *Blastocystis* species introduces uncertainty around its clinical significance. Further studies are required to investigate the pathogenicity of *Blastocystis* species, including molecular investigations into the various genetic subtypes.

## Data Summary

No data were generated or reused for this case report.

## Introduction

*Blastocystis hominis* is an anaerobic intestinal protozoan commonly found in human intestines [1]. The parasite lacks a cell wall and has a membrane-bound central body and mitochondria characteristic of protozoan morphology [2]. There are four reported distinct morphological forms, including cystic, amoeboid, granular and vacuolar phases [1, 3, 4]. The thicker surface coat of the cystic form is thought to be responsible for the development of resistance against harsh environments and anti-protozoal medications, as well as autoinfection [3, 5]. There are 13 reported subtypes of the parasite, with subtypes 1 and 3 being predominantly found in humans [6, 7].

*B. hominis* is distributed worldwide, with a reported prevalence of 1.4–20% in developed countries and up to 60% in developing countries [8, 9]. One study demonstrated that 8% of stool samples submitted to a Canadian reference laboratory were positive for *Blastocystis* species [10]. The proposed mode of transmission is faecal-oral through contaminated food and water [1, 9]. Some reported risk factors for the acquisition and infection with *B. hominis* include a history of travel to tropical areas, consumption of untreated water, poor nutrition, concomitant infections, immunosuppression and diabetes [4, 5, 11].

*B. hominis*’ pathogenicity in humans remains controversial; most people are believed to be asymptomatic carriers [12]. Some studies have demonstrated no correlation between gastrointestinal symptoms and the detection of *B. hominis* in stools [9, 13]. Conversely, multiple case studies have identified *B. hominis* as a possible pathogen through a presumed diagnosis of exclusion, which was further supported by patients having symptomatic improvement after eradication of the organism [1, 3–6, 11, 12, 14–20]. In symptomatic patients, clinical manifestations are typically mild and include nausea, anorexia, loose stools, abdominal cramps, flatulence, urticarial rashes and anal pruritus [1, 15, 16]. *B. hominis* has also been implicated in cases with more severe presentations, including appendiceal perforation, megacolon and rectal bleeding with ulcer formation [4, 5, 12, 15]. In immunocompromised patients, *B. hominis* has been reported to be an opportunistic pathogen and may cause chronic diarrhoea in patients living with the human immunodeficiency virus (HIV) [5]. It has also been reported to be the most commonly isolated intestinal parasite amongst patients receiving chemotherapy or prolonged dialysis, although it was unclear whether the patients were symptomatic [21].

There are no established diagnostic criteria for *B. hominis* infection, although typical investigations include light microscopic evaluation of stools, stool cultures, serologies and PCR [20]. Multiple previous studies have suggested a presumptive diagnosis of *B. hominis* infection when there are at least five organisms per high-power field present in the preparations of fresh stool of symptomatic patients [12]. The vacuolar form of the parasite is stained by iron haematoxylin and trichrome stain and is most commonly found in faecal specimens [3]. Amplification of *Blastocystis* DNA via PCR is also possible and enables identification of the *B. hominis* subtype [7]. Other laboratory markers associated with blastocystosis are faecal leukocytosis and rarely peripheral eosinophilia [4, 5, 20]. Some studies have previously suggested that *B. hominis* infections should only be a diagnosis of exclusion [20].

We report the case of a previously healthy immunocompetent man who presented to the hospital with fever, visual hallucinations and preceding gastrointestinal symptoms and was found to have two hepatic abscesses. *Entamoeba* spp. and *Blastocystis* spp. were identified from the hepatic abscess fluid.

## Case presentation

A 65-year-old previously healthy man presented to the emergency department of a community hospital with fevers, visual hallucinations and a 3-day history of diarrhoea and vomiting that had resolved the day prior to his presentation. He was admitted for management of hyponatraemia and right lower extremity cellulitis.

The patient initially received 2 days of cefazolin 2 g IV q8h for his lower extremity cellulitis, but the antibiotics were broadened to piperacillin-tazobactam 3.375 g IV q6h as the patient had persistent leukocytosis and fevers up to 39.3 °C measured orally. Five days after admission, the patient developed bloody diarrhoea with an associated 35-point haemoglobin drop to 118 g l^−1^, tachycardia and rapid atrial flutter. Blood tests drawn at the time of his clinical deterioration demonstrated a white blood cell count of 14.1×10^9^ l^−1^ with neutrophil and eosinophil counts of 11.4×10^9^ l^−1^ and 0.3×10^9^ l^−1^, respectively, sodium of 129 mmol l^−1^, total bilirubin of 36 μmol l^−1^, mildly elevated liver enzymes and C-reactive protein greater than 200 mg l^−1^. He was transferred to a tertiary hospital for admission to the intensive care unit. The next day, his white blood cell count continued to increase to 17.9×10^9^ l^−1^, and he also began to have an elevated eosinophil count of 0.9×10^9^ l^−1^.

A computed tomography (CT) scan of the abdomen demonstrated two hepatic abscesses within the right lobe of the liver, with one measuring 7.4×5.5×7.5 cm and the other measuring 6.5×5.9×6.7 cm. The Infectious Diseases service was consulted, and further exposure history revealed that the patient recently returned from an 8-month vacation in Cabo San Lucas, Mexico. During his time in Mexico, he stayed at a resort and avoided contaminated sources of food or other known infectious exposures except for raw seafood and alcoholic drinks with ice in the week preceding his return. Piperacillin-tazobactam was discontinued after 3 days and was subsequently transitioned to ceftriaxone 2 g IV q24h and metronidazole 750 mg IV q8h.

Stool testing for ova and parasites identified *Entamoeba* spp. via morphology and later confirmed to be *Entamoeba histolytica* by ELISA antigen testing. Moderate amounts of *Blastocystis* spp. were also identified in the same stool sample by microscopic examination of wet mount preparations and iron haematoxylin-stained smears. Additionally, stool specimens were positive for *E. histolytica* on the bioMérieux Biofire*^®^* gastrointestinal panel (Marcy-l'Étoile, France). The patient underwent ultrasound-guided percutaneous drainage of one of the hepatic abscesses under aseptic technique through a subcostal approach after receiving 1 week of antibiotics in total. Frank pus was described to have been aspirated. The Gram stain demonstrated no polymorphonuclear cells and no organisms seen. Bacterial culture of the hepatic aspirate was negative after 5 days of incubation. Ova and parasite examination of the hepatic aspirate on the wet mount identified *Entamoeba* spp. trophozoites in addition to many *Blastocystis* spp. cysts. The *Entamoeba* spp. was confirmed to be *E. histolytica* through nucleic acid amplification testing (NAT) performed on the hepatic aspirate; no confirmatory testing for *Blastocystis* spp. was performed. It is important to note that stool microscopy cannot differentiate between *Blastocystis* species. Laboratory analysis of the samples was completed within the regional laboratory in the admitting hospital, and results were confirmed at the reference laboratory by a parasitology expert. Other infectious work-up, including blood cultures, malaria NAT, *Strongyloides* serology and HIV serology, were negative.

He received 6 days of ceftriaxone in total before it was discontinued given that the ova and parasite examination was significant for *Entamoeba* spp*.* and *Blastocystis* spp. The patient clinically stabilized with no further symptoms and was transferred to the Internal Medicine Clinical Teaching Unit with a plan to complete 10 days of metronidazole in total, followed by 7 days of paromomycin 25–35 mg kg⁻¹ day⁻¹ orally divided three times daily to target any remaining luminal *E. histolytica* cysts. At this point, the patient had received a total of 11 days of empiric antimicrobials. A CT scan repeated 1 week later demonstrated interval enlargement of both hepatic abscesses from 7.4×5.5 cm to 10×9 cm and 6.5×5.9 cm to 10×7.5 cm, and the development of a new pelvic collection. The patient was additionally started on trimethoprim-sulfamethoxazole 130 mg/650 mg IV q6h by the Internal Medicine team in response to the progression of the abscesses with the aim of further broadening antimicrobial coverage, in the context of diagnostic uncertainty and limited literature suggesting possible pathogenic potential of *Blastocystis* spp. [7, 8]. Metronidazole was extended to ensure coverage for the *E. histolytica* because of inadequate source control with the progression of the hepatic abscesses. He also underwent an ultrasound-guided insertion of a second percutaneous drain. Repeat fluid cultures from the second drainage grew *Staphylococcus epidermidis* in broth only, which was thought to be a contaminant. A repeat ova and parasite examination of the hepatic aspirate 12 days later demonstrated *E. histolytica* only.

Upper endoscopy and colonoscopy were also performed which demonstrated multiple ulcers in the stomach, cecum and ascending colon. Biopsies taken from the colonic mucosa identified extensive acute colitis with ulceration and chronic inflammation, but no organisms were identified. Additionally, the patient was found to have positive IgG serology for *Helicobacter pylori*, and he was started on oral amoxicillin 1 g orally twice daily, clarithromycin 500 mg orally twice daily and bismuth subsalicylate in addition to the metronidazole he was receiving to complete quadruple therapy for *H. pylori* infection. Test of cure with stool antigen testing performed 2 weeks later was non-reactive.

In total, the patient received 3 weeks of metronidazole and trimethoprim-sulfamethoxazole, and he also completed a 1-week course of paromomycin. He was eventually discharged to the Hospital at Home programme where he had daily follow-ups with a nurse and a general internist for monitoring of the hepatic drains. Two days after discharge from the hospital ward, while under the care of the Hospital at Home programme, he developed one episode of hypotension with a systolic blood pressure of 72 mmHg and subjective fevers, and he was started empirically on ceftriaxone 2 g IV q24h. Ceftriaxone was discontinued after 4 days following negative blood cultures, and the patient remained clinically stable thereafter. He underwent multiple procedures to reposition and/or replace the hepatic abscess drains in the outpatient setting, and a repeat ultrasound 5 months later demonstrated a reduction in the size of the abscesses to 5.0×4.3×4.0 cm and 2.9×2.6×2.3 cm following removal of the drains.

## Discussion

Amoebic liver abscesses are a known extra-intestinal manifestation of invasive amoebiasis, which is caused by the protozoan *E. histolytica* following invasion of the intestinal mucosa and deposition in the liver through portal circulation [22]. Hu *et al*. reported on a case of a 63-year-old immunocompetent man who presented with bloody diarrhoea and fever and was found to have a hepatic abscess [23]. Stool and hepatic abscess fluid microscopy identified *Blastocystis* spp. only. However, based on positive PCR findings for *E. histolytica* in the abscess aspirate, the authors concluded that the likely infective organism was *E. histolytica*, and *Blastocystis* spp. was a secondary pathogen given the uncertainty of its pathogenicity [23]. No other reports of *Blastocystis* spp. being the sole pathogen identified in liver abscesses currently exist in the literature; this case report is the second case in which *Blastocystis* spp. was identified in liver aspirate fluid in conjunction with *E. histolytica*.

Evidence supporting a pathogenic role for *B. hominis* is mostly derived from case reports and observational studies (Table 1), which makes it difficult to draw firm conclusions about its pathogenicity. Symptomatic patients may have alternative explanations for their symptoms [14], and while many case reports attempted to exclude other potential infectious and non-infectious causes of the presenting symptoms, stool microscopy was usually the only test performed. The absence of confirmatory molecular or stool antigen testing limits the sensitivity of detecting other known enteric pathogens. In some case reports, authors suggested that the likely causative pathogen was *Blastocystis* spp., as patients had symptomatic improvement after treatment with metronidazole. However, clinical response does not establish *Blastocystis* spp. as the cause of symptoms because metronidazole also has activity against other common enteric pathogens. Furthermore, Miller and Minshew described 11 patients with enteritis in which *B. hominis* was identified in the stool, but there was no correlation between the resolution of their symptoms and the receipt of anti-protozoal treatment or eradication of *B. hominis* from the stools [24]. Some presentations described may have also been self-limited and may have resolved following their natural time course regardless of initiation of treatment.

**Table 1.** Suspected pathogenic cases of *Blastocystis* spp. found in the literature

Case study	Patient demographics	Clinical presentation	Significant investigations	Treatment	Outcome	Likelihood of pathogenicity
Yellanthoor	13-year-old male Healthy India	2-day history of acute watery diarrhoea (5 to 6 times per day), fever of 39.4 °C, abdominal cramps, blood-streaked stools	**Labs**: WBC 19.6×10^9^ l^−1^, 75% neutrophils **Stool microscopy**: numerous cystic and amoeboid forms of *Blastocystis* spp. **Stool culture**: negative	Failed third-generation cephalosporin×48–72 h Started on metronidazole	Symptoms resolved within 24 h of starting metronidazole	**Possible** – reportedly high parasite burden with *Blastocystis* spp., attempted to rule out other infective causes with stool culture, resolution of symptoms after empiric treatment with metronidazole
Janarthanan *et al.*	47-year-old male Healthy Detroit	3-week history of rectal bleeding. Had 2-week history of watery diarrhoea, abdominal bloating and pain that resolved spontaneously 4 weeks prior	**Colonoscopy**: large ulcers in the cecum, hepatic flexure and transverse colon with normal surrounding mucosa; small shallow ulcers in the rectum (may be consistent with *E. histolytica*) **Intestinal biopsies**: exudates with necrosis, inflammation, cryptitis, parasite seen **Stool microscopy with trichrome stain**: *Blastocystis* spp.	Treated with metronidazole×10 days	Symptoms resolved, no recurrence	**Unlikely** – possible infection with *E. histolytica* given presence of shallow rectal ulcers, did not perform stool culture or PCR testing for other causes of infectious diarrhoea, had previous episode of gastrointestinal symptoms that resolved spontaneously without treatment
Pedro-Botet *et al.*	32-year-old male Healthy Spain	5-day history of abdominal cramps and watery diarrhoea	**Labs**: WBC 7×10^9^ l^−1^, 47% neutrophils **Stool microscopy with Wright’s stain**: eosinophils, *Blastocystis* spp*.* (confirmed by trichrome stain) **Stool culture**: negative for *Shigella*, *Yersinia*, *Campylobacter*, *Salmonella*, *Aeromonas* **Enzyme immunoassay**: negative for rotavirus	Treated with metronidazole 500 mg QID×7 days	Symptoms resolved, asymptomatic after 5 months Follow-up stool exam negative for *Blastocystis* spp*.*	**Possible** – serial stool microscopy exams were positive for *Blastocystis* spp*.* only, pursued higher sensitivity testing with enzyme immunoassay for rotavirus, stool cultures were negative
Montero *et al.*	9-year-old male History of resected congenital right flank venous malformation Spain	2-day history of intermittent hypogastric abdominal pain, emesis, temperature of 37.4 °C	**Labs**: eosinophilia, elevated CRP on day 5 post-operative routine blood work **Peritoneal fluid culture**: *Escherichia coli*, *Streptococcus anginosus*, *Fusobacterium necrophorum*, *Bacteroides fragilis* and *Bacteroides distasonis* **Stool microscopy**: *Blastocystis* spp*.* cysts **Stool culture**: negative **Haematoxylin and eosin and Giemsa stains**: *Blastocystis* spp*.* cysts in the appendix	Single-incision laparoscopic appendectomy Meropenem 80 mg kg^−1^×7 days based on surgical findings Metronidazole×10 days after *Blastocystis* spp*.* seen in stool studies	Symptoms resolved, remained asymptomatic Repeat stool study after 10 days of metronidazole showed resolved parasitosis	**Unclear** – patient was asymptomatic but had laboratory markers of eosinophilia and elevated CRP that was detected incidentally; stool cultures were negative
Andiran *et al.*	**Case 1** 12-year-old male Turkey	3-day history of right lower quadrant pain, bilious vomiting, diarrhoea, fever of 38.6 °C	**Labs**: WBC 19.2×10^9^ l^−1^, CRP 59 mg l^−1^ **Stool microscopy**: ≥5 *Blastocystis* spp*.* per high power field, ‘Negative markers’ for rotavirus	Metronidazole, TMP/SMX	Recovered	**Unclear** – did not perform stool culture or PCR testing for other causes of infectious diarrhoea, resolution of symptoms after empiric treatment with metronidazole and TMP/SMX
	**Case 2** 11-year-old male Turkey	2- to 3-day history of abdominal pain, nausea, vomiting, fever, watery stools, fever of 38.5 °C	**Labs**: WBC 16.2×10^9^ l^−1^, CRP 77 mg l^−1^ Salmonella-Brucella agglutination test negative **Stool microscopy**: vacuolar forms of *Blastocystis* spp*.*	Metronidazole, TMP/SMX	Recovered	**Unclear** – did not perform stool culture or PCR testing for other causes of infectious diarrhoea, resolution of symptoms after empiric treatment with metronidazole and TMP/SMX
Lucia *et al.*	67-year-old male History of haemophilia A, chronic hepatitis C Spain	6-week history of watery diarrhoea with increasing bloody rectal discharge, colicky abdominal pain, flatulence, tenesmus	**Colonoscopy**: generalized oedema, erythema, ulcers **Stool culture and *Clostridium difficile* toxin**: negative Repeat stool microscopy in 4 weeks showed vacuolar forms of *Blastocystis* spp.	Metronidazole 500 mg TID×10 days	Improvement in symptoms within 2 days Repeat stool microscopy negative 4 weeks after stopping metronidazole	**Unclear** – symptoms already resolving prior to treatment with metronidazole, work-up for other causes with stool culture and *C. difficile* toxin testing were negative
Carrascosa *et al.*	76-year-old male Spain	Generalized abdominal cramps, bloody diarrhoea	**Stool microscopy**: faecal leukocytes, 30 *Blastocystis* spp*.* per high power field **Stool cultures**: negative for *C. difficile*, *Salmonella*, *Shigella*, *Yersinia*, *Campylobacter* and *Vibrio* **Sigmoidoscopy**: diffuse erythema **Rectal and sigmoid biopsies**: acute inflammation Unremarkable mesenteric/coeliac arteriography, barium enema, abdominal ultrasound	Metronidazole 750 mg TID×7 days	Symptoms resolved Repeat stool microscopy 2 and 7 weeks after treatment was negative	**Possible** – reportedly high parasite burden with *Blastocystis* spp*.*, work-up for other causes with stool culture was negative, resolution of symptoms after empiric treatment with metronidazole
Lee *et al.*	29-year-old female Rheumatoid arthritis – started on prednisone 15 mg daily Jamaica	3 weeks of diarrhoea (3 to 4 times per day) with occasional blood and mucous, lower abdominal pain, vomiting, fever of 38.1 °C Increased knee pain and swelling	**Labs**: WBC 8.1×10^9^ l^−1^, 62% neutrophils and 2% eosinophils, ESR 150 mm in first hour **Sigmoidoscopy**: mild to moderate erythema/oedema **Blood, synovial fluid and urine cultures**: negative **Endocervical swab**: negative **Light microscopy and trichrome stain** of synovial fluid from left knee showed *Blastocystis* spp*.* **Stool microscopy**: >5 *Blastocystis* spp*.* per high power field **Stool cultures**: negative for *Salmonella*, *Shigella*, *Campylobacter*, other parasites	No improvement on 5 days of ceftriaxone Switched to metronidazole 400 mg q8h×2 weeks	Abdominal symptoms and left knee pain resolved over 5 to 7 days Repeat knee aspirate and stools negative for *Blastocystis* spp*.*	**Possible –** detection of *Blastocystis* spp*.* in synovial fluid, which is normally sterile, possible dissemination in the case of immunosuppression, clinical response of both diarrhoea and knee inflammation to metronidazole
Al-Tawil *et al.*	4-year-old female Healthy USA	3-week history of rectal bleeding, progressed to 5 to 6 watery stools per day with blood clots, fever of 38 °C Had UTI and was treated with Septra prior to presentation for persistent rectal bleeding	**Labs**: WBC 10×10^9^ l^−1^ **Endoscopy**: normal **Colonoscopy**: diffuse superficial ulcers with pseudomembranes throughout colon **Histologic exam**: moderate inflammation with eosinophils, *Blastocystis* spp*.* in the superficial lamina propria **Stool cultures**: negative for *Salmonella*, *Shigella*, *Yersinia*, *Campylobacter,* negative for viral pathogens and *C. difficile* toxin **Stool microscopy**: 2 to 3 *Blastocystis* spp*.* cysts per high-power field	Metronidazole 10 mg kg^−1^ per dose q6h	Symptoms resolved after 1 week of treatment	**Unclear** – was previously treated with TMP/SMX prior to stool testing with no clinical response at all despite activity against *Blastocystis* spp*.*, work-up for other causes with stool culture was negative
Salvi *et al.*	86-year-old female History of stroke with neurological deficits in the lower extremities Italy	2-week history of abdominal distention, bilious vomiting, diarrhoea	**Labs**: WBC 6.3×10^9^ l^−1^, 86% neutrophils **X-ray**: distended loops of large bowel **Stool culture**: negative ***C. difficile* toxin**: negative **Stool microscopy**: >10 *Blastocystis* spp*.* cysts per high-power field **Biopsies of upper rectum**: mild fibrosis of the mucosal lamina propria	Metronidazole 500 mg TID IV×10 days TMP/SMX 480 mg q6h	Initially improved on antibiotics but abdomen became distended again after 9 days of treatment, found to have recurrent megacolon and diarrhoea, then underwent cecostomy	**Unclear** – work-up for other causes with stool culture and *C. difficile* was negative, reportedly high parasite burden with *Blastocystis* spp., clinical worsening despite targeted treatment against *Blastocystis* spp*.*
Levy *et al.*	74-year-old male History of non-insulin-dependent diabetes, hypertension, aortic stenosis with aortic valve replacement Israel	1-week history of diarrhoea up to five times per day, colicky abdominal pain, nausea, fever of 38 °C	**Labs**: WBC 30.1×10^9^ l^−1^, 90% neutrophils, 6% lymphocytes, 4% monocytes **Blood cultures**: negative **Stool cultures**: negative **C. difficile toxin**: negative **Stool microscopy**: *Blastocystis* spp.	Metronidazole 2 g IV per day	Symptoms improved in 10 days	**Unclear** – work-up for other causes with stool culture and *C. difficile* was negative, resolution of symptoms after empiric treatment with metronidazole
Tai *et al.*	6 patients (3 female and 3 male), average age 38.7 years old Relapsing ulcerative colitis diagnosed by clinical, radiological, colonoscopic and histologic criteria	8 to 26 months of blood and purulent stool	**Stool microscopy:** 6 patients’ samples positive for *Blastocystis* spp*.* – only included patients who had four serial stool exams positive for *Blastocystis* spp*.* only	Metronidazole 910 mg BID×10 to 14 days	Symptoms resolved 3 weeks later Repeat stool microscopy and colonoscopy were negative	**Unclear** – only selected for patients who had serial positive stool exams with *Blastocystis* spp*.* only, did not perform stool culture or PCR testing for other causes of infectious diarrhoea, resolution of symptoms after empiric treatment with metronidazole
Basak *et al.*	22-year-old female Healthy India	6-month history of alternating diarrhoea and constipation, recent passage of fresh stool	**Stool microscopy**: Gram stain positive for vacuolar forms of *Blastocystis* spp*.* **Stool culture on Lowenstein–Jensen medium**: *B. hominis* **Stool culture**: *E. coli*	Symptoms not responsive to treatment with TMP/SMX, furazolidone, norfloxacin, ciprofloxacin, antacids Switched to metronidazole 400 mg TID×7 days	Symptoms resolved on follow-up after metronidazole	**Unlikely** – *E. coli* also isolated, treated with TMP/SMX prior to stool testing with no clinical response at all despite activity against *B. hominis*
Santos *et al.*	22-year-old female Brazil	Localized pain in left hypochondrium initially, acute worsening of pain 3 years later	**CT abdomen**: two splenic cysts (11×11 cm) **Urine culture**: positive for *Streptococcus agalactiae* **Splenic cyst fluid analysis**: histiocytes, lymphocytes, polymorphonuclear leukocytes, lysed erythrocytes, *B. hominis* (confirmed by PCR)	Laparoscopic excision of splenic cyst	Recovered well, discharged 2 days after operation	**Unclear** – performed confirmatory testing with PCR testing, also numerous possible non-infectious causes of splenic cysts

CRP, C-reactive protein; TMP/SMX, trimethoprim/sulfamethoxazole; WBC, white blood cell.

Other studies have concluded that there is no association of *B. hominis* with symptomatic infections [9, 13] and that the parasite is only pathogenic in cases of immunosuppression [19]. A study by Leder *et al*. suggested that there may be possible carriage of *B. hominis*, as a higher proportion of patients who previously tested positive for *B. hominis* in the stools continued to test positive when they were symptomatic compared to those with baseline negative tests [9].

Some studies suggest that different genetic subtypes of *Blastocystis* spp*.* may be responsible for the varying degrees of pathogenicity observed, with subtypes 1 and 3 being more pathogenic than the others [7]. One case study identified subtype 3 of *Blastocystis* spp*.* from the fluid of a splenic cyst through microscopic examination and PCR, although the authors were uncertain about the exact pathogenic mechanism [6]. The pathogenicity of various strains and subtypes of *Blastocystis* spp*.* warrants further investigation, as genomic analysis was not commonly reported in case studies attributing *B. hominis* as the causative organism.

In this case study, the clinical picture is most consistent with amoebic liver abscesses secondary to *E. histolytica*. When the patient had refractory disease with enlargement of the liver abscesses while on metronidazole, the repeat microscopic examination of the aspirate fluid identified only *E. histolytica* and not *Blastocystis* spp*.*, adding to the evidence that the primary pathogen was likely *E. histolytica*. Another possibility for this finding would be that *Blastocystis* spp*.* was more easily treated and successfully cleared compared to *E. histolytica.* Detection of *Blastocystis* spp*.* in this case was only performed morphologically, and we are unable to speciate the organism without PCR testing. Contamination of the hepatic aspirate can also be considered, although given that moderate numbers of *Blastocystis* organisms were seen on the wet mount, and as the hepatic aspirate was obtained percutaneously with aseptic technique, contamination during specimen collection, handling and processing is unlikely.

The clinical significance of detecting *B. hominis* in human samples remains unclear, leading to uncertainty over whether patients should be treated for suspected *B. hominis* infections. Treatment of *B. hominis* typically consists of a 10- to 14-day course of anti-protozoal medications, most commonly metronidazole. Treatment may be warranted for immunocompromised patients or those with more severe gastrointestinal symptoms when no other causes are identified [5, 16]. However, given metronidazole’s broad spectrum of activity, patients may inadvertently be treated for another organism that is more likely responsible for their symptoms. In this case report, the patient received a prolonged 3-week course of metronidazole and trimethoprim-sulfamethoxazole with coverage against both *E. histolytica* and *Blastocystis* spp*.* that were identified in the hepatic abscess fluid. While there was initial symptomatic improvement, the abscesses progressed in size, likely secondary to inadequate source control, which ultimately necessitated further management of the hepatic drains before resolution of his clinical symptoms.

## Conclusion

To our knowledge, this is the second reported case of hepatic abscesses in which *Blastocystis* spp. were identified along with *E. histolytica*. While there is ongoing uncertainty of the pathogenicity of *Blastocystis* spp*.* as an intestinal parasite, this case offers some insight into its host interactions. Further analytical and molecular studies on the various subtypes of *Blastocystis* spp*.* are required to provide further evidence on its role in the human microbiome and its potential pathogenicity.

## Abbreviations

CT: computed tomography
HIV: human immunodeficiency virus
NAT: nucleic acid amplification testing
